# A new era of inequality: profound changes to mortality in England, Scotland, and 10 major British cities

**DOI:** 10.1093/eurpub/ckaf008

**Published:** 2025-02-19

**Authors:** Maria Teresa de Haro Moro, Lauren Schofield, Rosalia Munoz-Arroyo, Gerry McCartney, David Walsh

**Affiliations:** Public Health Scotland, Edinburgh, United Kingdom; Public Health Scotland, Edinburgh, United Kingdom; Public Health Scotland, Edinburgh, United Kingdom; School of Social and Political Sciences, University of Glasgow, Glasgow, United Kingdom; School of Health and Wellbeing, University of Glasgow, Glasgow, United Kingdom

## Abstract

Deeply concerning changes to UK health trends have been noted since the early 2010s, including a widening of mortality inequalities. Given the importance of urban areas to national health outcomes, we sought to address gaps in the evidence by examining trends in intra-city mortality inequalities across Britain, including assessing the impact of the peak COVID-19 pandemic period. Age-standardized mortality rates were calculated (for England, Scotland, and 10 major UK cities) by age (all ages, 0–64 years), sex, year (1981–2020), and country-specific and city-specific area-based quintiles of socio-economic deprivation. Trends in absolute and relative inequalities in mortality by country and city were analysed by means of the Slope Index of Inequality (SII) and the Relative Index of Inequality (RII), respectively. Profound changes to mortality trends and inequalities were observed across both nations and all cities in the decade up to 2020, including increases in death rates among the 20% most deprived populations of almost every city. For deaths at all ages, this was particularly evident in Leeds, Liverpool, Edinburgh, Dundee, and Glasgow. For 0–64 years, Scottish cities stood out. With few exceptions, both absolute and relative inequalities increased in the same time period. COVID-19 further increased death rates and inequalities. The analyses provide a hugely concerning picture of worsening mortality and widening inequalities across England and Scotland. When viewed in the context of the evidence for the impact of UK government austerity policies on population health, they represent a wake-up call for both current and future UK governments.

## Introduction

Deeply concerning changes to a variety of population health measures have been noted in the UK since the start of the last decade. These include worsening trends in birth outcomes among poorer populations [1], worsening mental health among particular age groups [2], and adverse changes to mortality and related indicators. The latter include an overall stalling of previous (decades long) improvement, increasing death rates in more socioeconomically deprived areas, and a subsequent widening of inequalities [3–5]. These changes predate the COVID-19 pandemic but have been exacerbated by it [6].

Evidence of changes to mortality rates and inequalities in the UK has thus far been shown at nation level (England, Scotland, Wales, and Northern Ireland [3, 4], or in analyses of large population groups within those nations [3–5, 7–9]). The contribution of urban areas to national level health outcomes is well understood: This relates to multiple factors including increased urbanisation (meaning that greater proportions of national populations live in urban areas), the adverse effects of built environments (e.g. housing quality and density, air pollution) on both physical and mental health, and—crucially for an understanding of health outcomes and inequalities in the UK—the fact that poverty levels across the nations of the UK are notably higher in urban areas [10]. However, while analyses of changes to intra-city mortality inequalities have been undertaken, this has only been done systematically for Scottish UK cities [3, 6]. Here we seek to address gaps in the evidence base by: Examining intra-city mortality inequalities across Britain (England and Scotland); and updating previous analyses to assess the impact of (some of) the pandemic period. The overall aim was therefore to provide an updated picture of changes to national and urban mortality inequalities across Great Britain in the 21st century.

## Methods

All-cause mortality data were obtained from the Office for National Statistics (ONS) for England, and from the National Records of Scotland (NRS) for Scotland. Data were broken down by year (1981–2020), 5-year age group, sex, UK nation (England, Scotland), city (listed below), and socioeconomic deprivation quintile (for 2001–2020 only) (discussed further below). Individual death records had deprivation quintiles assigned, based on postcodes; data were then aggregated accordingly. Matching population denominator data were obtained from the same data sources.

Area-based deprivation quintiles were derived from the English Index of Multiple Deprivation (IMD) [11] and the Scottish equivalent (Scottish Index of Multiple Deprivation—SIMD [12]). As previously described [3], despite some differences in composition and spatial scale of these two measures of area-based socioeconomic deprivation, the two indices are very similar and comparable: Further details are included in the Supplementary Material Box S1. Data were available from 2001 onwards and, following previous methodology [3, 6, 9], different versions of both regularly updated indices were used across the analytical time period (Table S2 in the Supplementary Material Appendix S1). Different sets of deprivation quintiles were derived: National quintiles (for England and, separately, Scotland) and city-specific quintiles (created separately for each city).

Ten cities across England and Scotland were included, each defined by current local authority boundaries. The cities were Birmingham, Bristol, Leeds, Liverpool, Manchester and Sheffield (England), and Aberdeen, Dundee, Edinburgh, and Glasgow (Scotland). These were chosen in relation to their size: Excluding London, these are the largest cities in their respective countries, with populations ranging from c.150 000 (Dundee) to c.635 000 (Glasgow) in Scotland, and from c.465 000 (Bristol) to c.1.1 million (Birmingham) in England (Table S1 in the Supplementary Material S1). London was excluded to enable more meaningful city comparisons: as has been previously outlined, London’s vastly different size and ethnic profile make such comparisons difficult to achieve [13].

For background to the main analyses, levels and inequalities in ‘income deprivation’ (the proportion of the total population in receipt of, or dependent on someone in receipt of, low income-related social security benefits—and one of the ‘data domains’ of both the IMD and SIMD) in each city were calculated, using the most recent version of the two deprivation indices highlighted above.

Age-standardized mortality rates (ASMRs) were calculated by year, UK nation, city, national and city-specific deprivation quintile, and sex, using the 2013 European Standard Population. Analyses were undertaken for both overall mortality (i.e. all ages) and premature mortality (defined as less than 65 years). Given the high levels of fluctuation of rates at city, and sub-city level, ASMRs are presented as 3-year rolling averages. We chose ASMRs, rather than life expectancy, both because we wished to include analyses of premature mortality, and because we were building on previous analytical work which used ASMRs [3]. Trends in absolute and relative inequalities in mortality were analysed by calculating the Slope Index of Inequality (SII) and the Relative Index of Inequality (RII), respectively, using the country-specific, and city-specific, area deprivation quintiles to rank the populations, and based on linear regression [14]. The SII calculates the absolute gap in mortality rates across quintiles, taking into account both the ASMR, and population size, of each quintile (i.e. not just those at the extremes (20% most and least deprived)). By dividing the SII by the ASMR of the whole population (country or city), we calculate a relative measure of inequality—the RII.

Patient and public involvement: neither patients nor the public were involved in the design of this study.

## Results

As background to the main analyses, analyses of ‘income deprivation’ in the most recent versions of the English and Scottish deprivation indices showed notably higher levels of such deprivation in Manchester, Birmingham, and Liverpool (approximately 22%–23% of the population classed as ‘income deprived’) compared with Bristol, Leeds, and Sheffield (14%–16%). In Scotland, Aberdeen and Edinburgh (9%) were notably less deprived than Dundee (16%) and Glasgow (19%) (Fig. S1). However, all cities showed considerable levels of inequality in this measure: For example, in the most deprived 20% of neighbourhoods in Bristol, over 30% of the population were classed as income deprived compared with just 3% in the least deprived areas; the equivalent figures for Liverpool were 41% and 5.5% (Fig. S2). Note that definitions of income deprivation differ slightly between the two indices (Table S3).

**Figure 1.**
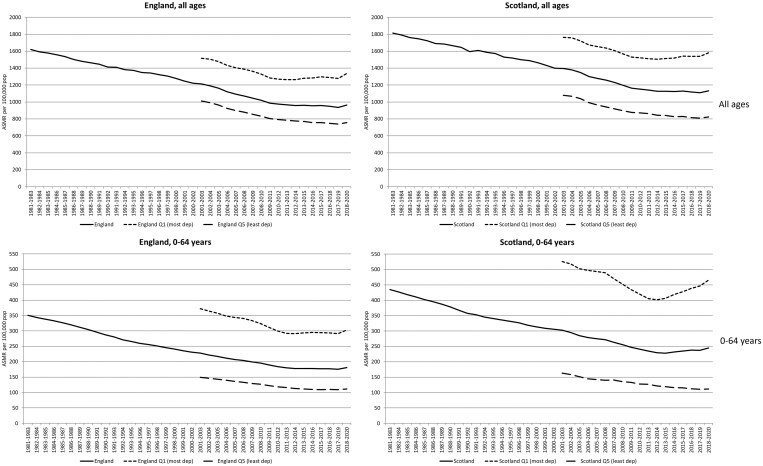
Age-standardized mortality rates per 100 000 population (3-year rolling averages), 1981–2020, all ages and 0–64 years: Scotland, England, and their 20% most and least deprived populations.

Figure 1 shows trends in ASMRs in Scotland and England for both all-age and premature mortality. Rates are for both sexes combined; equivalent charts for males and females separately are shown in Figs S3 and S4, respectively, in the Supplementary Material S1.

In all charts, a change in the long-term trends can be seen in the early part of the last decade: a ‘levelling off’ of previous improvement (e.g. England, all ages—especially females), or a reversal (worsening) of previous falling rates (e.g. most deprived quintiles of England (all ages) and Scotland (all ages and 0–64 years)). The changes are particularly dramatic for premature mortality in Scotland. The additional impact of the first year of the COVID-19 pandemic (2020) is also visible.

**Figure 2.**
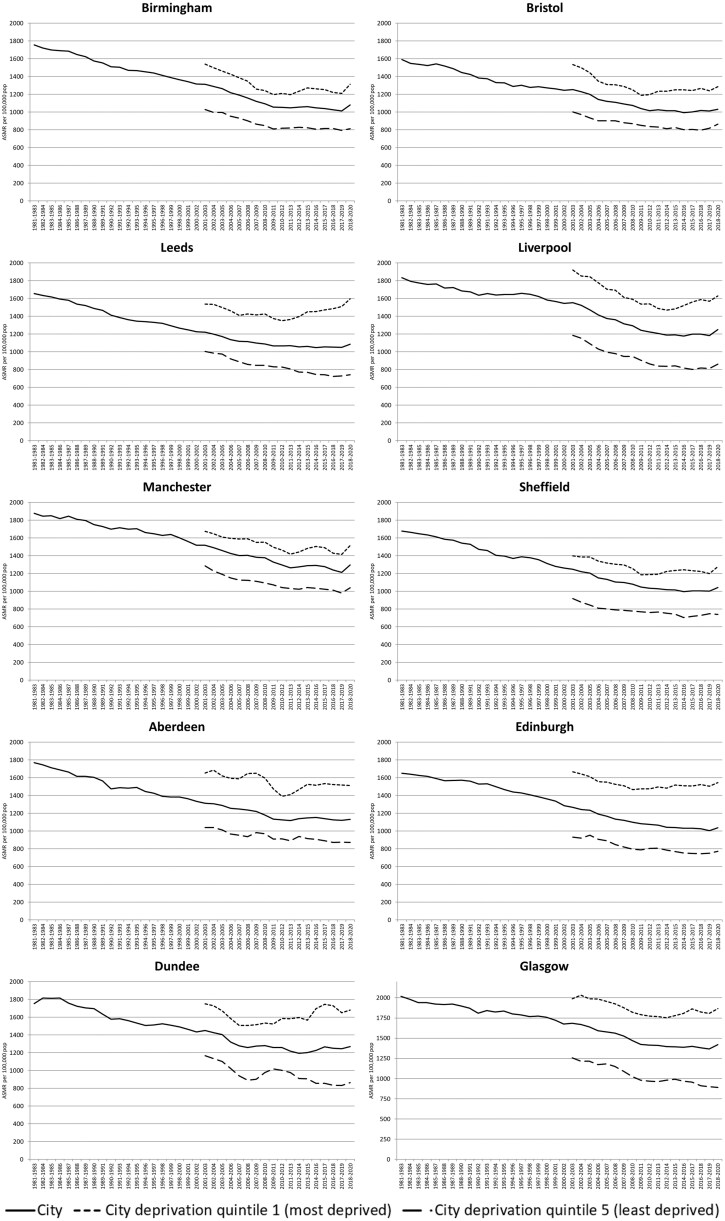
Age-standardized mortality rates per 100 000 population (3-year rolling averages), 1981–2020, all ages, for 10 British cities and their 20% most and least deprived populations.

**Figure 3.**
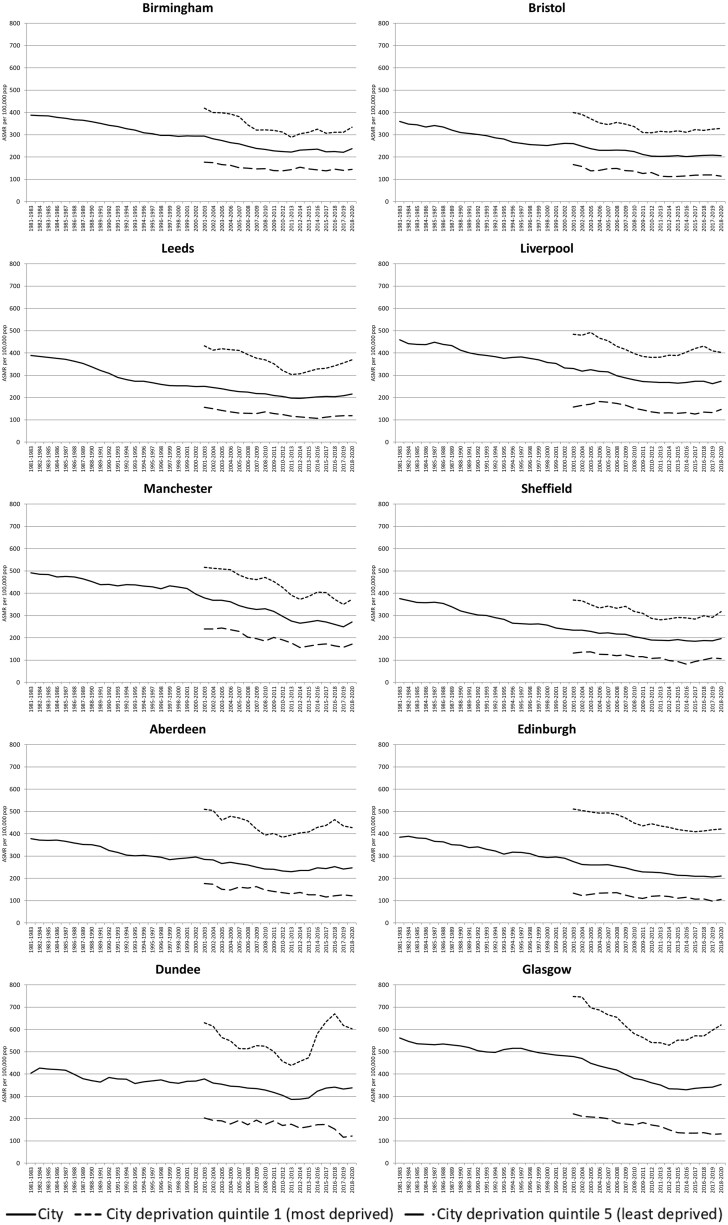
Age-standardized mortality rates per 100 000 population (3-year rolling averages), 1981–2020, 0–64 years, for 10 British cities and their 20% most and least deprived populations.

Figures 2 (all ages) and 3 (0–64 years) show the equivalent analyses for the 10 British cities. Again, this is for both sexes combined, with separate charts for males and females included in the Supplementary Material S1 (Figs S5–S8). Despite greater fluctuation in rates, especially in smaller cities such as Aberdeen and Dundee, a fairly consistent pattern is apparent in most cities and for both broad age groups: worsening mortality rates for the most deprived quintile of each city, and a resulting widening gap between the most and least deprived sets of areas. In Fig. 2, this is apparent in most cities, but especially in Leeds, Liverpool, Edinburgh, Dundee, and Glasgow. For premature mortality (Fig. 3), Leeds, Aberdeen, Dundee, and Glasgow stand out.

The sex-specific analyses show particularly dramatic changes to all-age female mortality rates in the most deprived quintiles of Leeds, Liverpool, and Edinburgh; changes to premature mortality rates are clearest in some of the Scottish cities.

However, overall, notable changes to mortality rates in the most deprived parts of all 10 cities are apparent to a greater or lesser degree.

**Figure 4.**
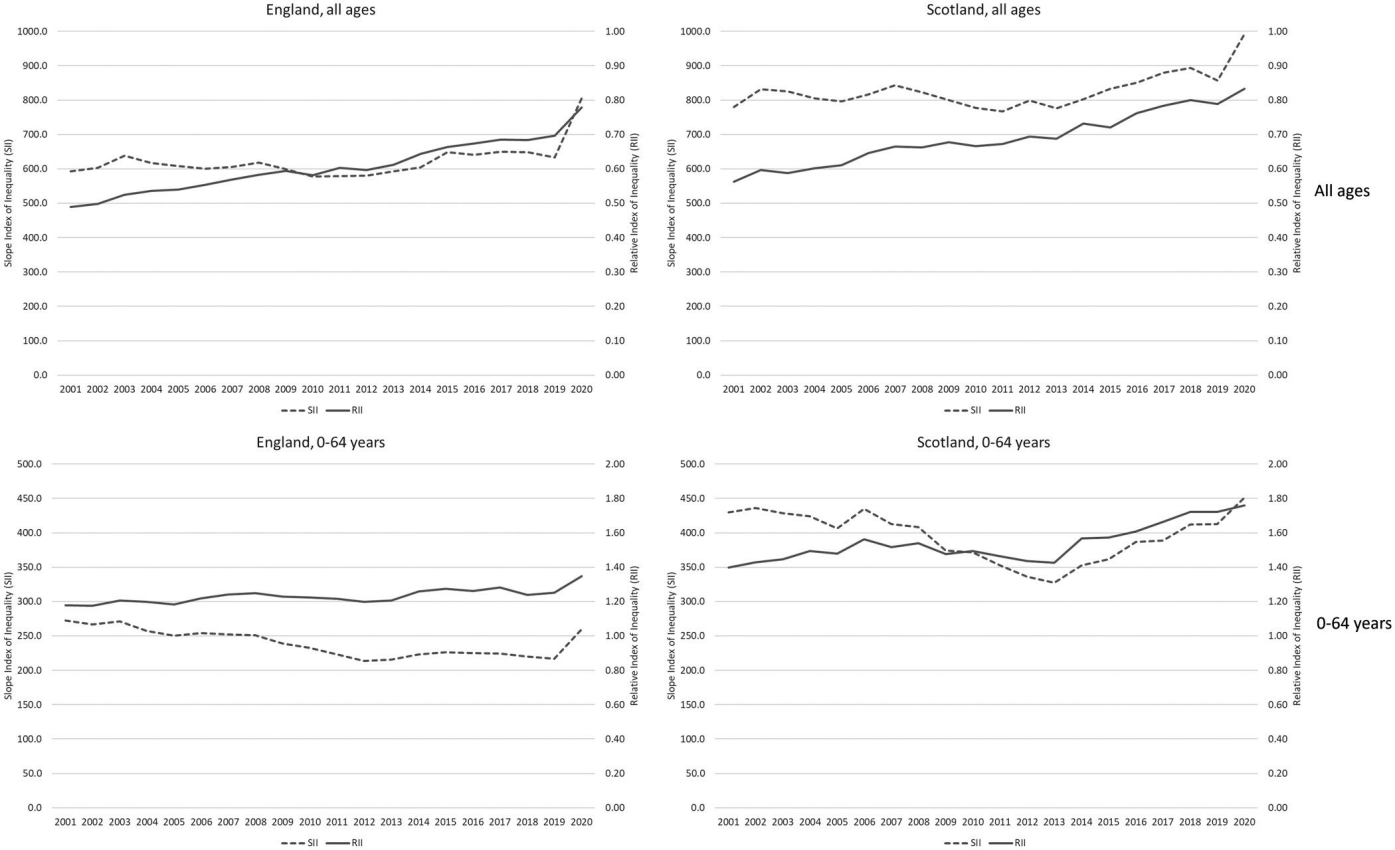
Trends in the Slope Index of Inequality (SII) and Relative Index of Inequality (RII) for age-standardized mortality rates by deprivation quintile, 1981–2020, all ages and 0–64 years, Scotland and England.

Figure 4 shows trends in absolute (SII) and relative (RII) inequalities in Scotland and England, for both all ages combined, and 0–64 years. Relative inequalities generally increased over the whole 20-year period analysed. In contrast, absolute inequalities generally decreased between 2000 and the early 2010s, but increased thereafter. Both measures of absolute inequality increased sharply in 2020, particularly for all ages combined. Overall inequalities were wider in Scotland than in England over the whole period.

Figure S7 in the Supplementary Material S1 presents trends in the same two measures of inequality for each British city. There is greater year-by-year fluctuation of values at city level; thus for ease of interpretation, the lines have been smoothed by the use of 3-year averages (however, values for each year are also included in the Supplementary Material Appendix S1 (Tables S4 and S5)). The results are broadly similar to those of the national level analyses shown in Fig. 4: with a small number of exceptions (e.g. Bristol), relative inequalities in mortality increased throughout the period, while trends in absolute levels of inequality were either flat or decreasing prior to the early 2010s, but increased notably thereafter. Inequalities were highest in the Scottish cities, as well as in Leeds and Liverpool.

Note finally that for clarity of presentation, Figs 1–4 do not include 95% confidence intervals (CIs); however, versions of these figures which do incorporate CIs are included in the Supplementary Material Appendix S1 (Figs S10–S14). As can be seen, for the ASMRs especially, the addition of the CIs does not change the principal findings. For the measures of inequality (SII and RII), however, the CIs can be wide, especially for the city-level analyses; thus, caution is required in interpreting some of those trends.

## Discussion

### Overall findings and implications

What emerges from the analyses shown here is a picture of widening mortality inequalities across England and Scotland and 10 major cities of these nations.

At the national level, we have seen—since the early 2010s when trends started to change—a reversal of previously declining mortality rates among more socioeconomically deprived populations, particularly among females of all ages, and for premature mortality in Scotland. Dramatic changes in mortality rates among such poorer populations have also been seen across multiple British cities, with a resulting widening of socioeconomic inequalities. Indeed, one can argue that since the early 2010s, Britain has entered a new era of health inequality. Previously, relative inequalities had widened because mortality rates of the less deprived populations were falling at a greater rate than those of more deprived populations; however, the latter were still declining. Now inequalities have widened—both in a relative and absolute sense—because mortality rates of poorer populations have actually increased. The data show very clearly that these changes predate the COVID-19 pandemic by many years.

### Relevance to other studies

The causes of these extraordinary changes to mortality rates and inequalities in Britain are increasingly well understood. A large evidence base has emerged in recent years, demonstrating that the changes have been caused in large part by UK government ‘austerity’ policies which were introduced from 2010 onwards, which are still in operation today, and which have had a devastating impact on the health—and mortality—of the poorest in UK society [15–17]. It has been estimated that austerity policies reduced UK Government spending by approximately £540 billion between 2010 and 2019 [18]. The two key components of austerity—cuts to social security payments and eligibility, and reductions in key social services brought about by cuts to local government funding—have particularly impacted on the poorest and most vulnerable in society [19, 20]. The causal pathways between such policies and population health are also well understood: cuts to individual income through reductions in social security have brought about increased levels of poverty, stress, poor mental health, and health-harming ‘coping mechanisms’ [21–24], while cuts to local services (e.g. housing, education) impact via a range of different social determinants of health [25]; cuts to health and social care services (including homelessness, alcohol and drugs, mental health) have also had direct effects [26, 27]. All of these have been demonstrated in a broad range of studies, with impacts demonstrated on outcomes of poor mental health [28–30], multi-morbidity [31], drugs harm [32–34], and overall mortality and life expectancy [16, 17, 35–37]. Of particular relevance to these city-based analyses is the fact that both cuts to social security and cuts to services have been greatest in more deprived areas, which tend to be largely urban (see the Supplementary Material Appendix S1 for additional references).

Across these and other studies, increased death rates (and/or reductions in life expectancy) have been shown for more deprived populations in all four nations of the UK, and in other poorer populations defined in different ways [4–9]. This tallies with the national- and city-level analyses shown here.

The precise timing of the changes to trends (both for more deprived populations, but also in terms of the associated stalling of improvement at country level) has been estimated using particular statistical techniques [9, 16]. Although the precise values of the ‘break points’ have differed slightly according to sex, age, and geography, they have tended to centre around c.2012—i.e. within 2 years of the start of the austerity period in the UK.

The greater increase in premature mortality rates among deprived populations in Scotland (including in individual Scottish cities) compared to England can be attributed in part to greater numbers of, and increases in, drug-related deaths in Scotland. Higher death rates historically are known to have been driven by a range of factors, including the influence of particularly vulnerable birth cohorts, the type of drugs on the market place, sophisticated supply systems, and relatively greater affordability over time [15, 38]. These have been made worse by austerity, with the impact of cuts to both personal income and the funding of services as important contributory factors; these have also been associated with increases in drug deaths elsewhere in the UK [15, 32–34].

Similar detrimental impacts of austerity policies have also been shown in other high-income countries [15–17], and also for a range of other outcomes such as child health and maternity-related events [1, 39].

Aside from the impact of austerity, the overall stalling of mortality rates in Scotland and England has also been shown to have been influenced—albeit to a relatively small degree—by prior increases in obesity in the population [40].

### Strengths and weaknesses

For the first time, we are able to show city-specific mortality inequalities across 10 major cities of Britain. We have quantified changes to inequalities both in absolute and relative terms. At country and city level, we have analysed trends over four decades and have been able to show the impact of (one year of) the COVID-19 pandemic on those rates. We have used data for the whole population—not samples—of Scotland and England and their major cities, using a robust population health indicator that is less susceptible to different biases known to affect self-reported measures of health. However, various limitations are also acknowledged: Only 1 year (2020) of the pandemic period is included, as ONS were unable to provide us with data for 2021 for the English cities (however, we know from previous analyses of the Scottish areas that mortality rates, and inequalities, increased further in 2021 [6], and this is therefore also likely to be true of the English cities); we were only able to access (and thereby analyse) data for all causes of death combined, and not individual causes of death (and thus we are unable to compare the relative contributions of particular causes to the overall mortality changes in each location); deprivation data were only available from 2001; the study is purely descriptive, and we did not seek to estimate break points in trends; and we were limited to only two very broad age groups.

## Conclusions

The analyses provide a hugely concerning picture of worsening mortality rates for many, and widening inequalities for all, across England, Scotland, and—with the exception of London—the countries’ main urban areas. When viewed in the context of the considerable evidence base for the devastating impact of UK government austerity policies on population health measures, they should act as a wake-up call for both current and future UK governments.

## Supplementary Material

ckaf008_Supplementary_Data

## Data Availability

There are no new data associated with this article. Key points For the first time we analyse city-specific mortality inequalities across ten major British cities. We demonstrate a consistent and concerning widening of urban mortality inequalities since the early 2010s, made worse by the COVID-19 pandemic. We quantify changes in both absolute and relative inequalities in mortality at British city and nation level. When viewed in the context of the large evidence base for the devastating impact of UK Government austerity policies on population health measures, the analyses should act as a wake-up call for both current and future UK governments.

## Notes

**EUPHA Membership:** David Walsh, Membership No. 12672
