# Supplementary material for: A new era of inequality: profound changes to mortality in England, Scotland, and 10 major British cities
Source: Eur J Public Health. 2025 Feb 19;35(2):235–41. doi: 10.1093/eurpub/ckaf008 (PMC11967883; doi:10.1093/eurpub/ckaf008)
Supplement: ckaf008_Supplementary_Data [file ckaf008_supplementary_data.docx]

**Supplementary material**

**Table A1. Cities’ population size (2020)^[[1]](#footnote-1)^**

| **Country** | **City** | **Total population at 2020** |
| --- | --- | --- |
| Scotland | Aberdeen | 229,060 |
|  | Edinburgh | 527,620 |
|  | Dundee | 148,820 |
|  | Glasgow | 635,640 |
|  |  |  |
| England | Birmingham | 1,140,525 |
|  | Bristol | 465,866 |
|  | Leeds | 798,786 |
|  | Liverpool | 500,474 |
|  | Manchester | 555,741 |
|  | Sheffield | 589,214 |

*Note: as stated in the main paper, cities are defined by local authority boundaries.*

**Table A2. Versions of the Scottish and English Indices of Multiple Deprivation (SIMD and (E)IMD) used in the analyses.**

| Scottish Index of Multiple Deprivation (SIMD) | |  | (English) Index of Multiple Deprivation (IMD) | |
| --- | --- | --- | --- | --- |
| Version | Analysis years |  | Version | Analysis years |
| SIMD 2004 | 2001-04 |  | IMD 2004 | 2001-05 |
| SIMD 2006 | 2005-07 |  | IMD 2007 | 2006-08 |
| SIMD 2009 | 2008-10 |  | IMD 2010 | 2009-13 |
| SIMD 2012 | 2011-13 |  | IMD 2015 | 2014-16 |
| SIMD 2016 | 2014-17 |  | IMD 2019 | 2017-20 |
| SIMD 2020 | 2018-20 |  |  |  |

*Note that while the spatial units on which the deprivation indices are based can move between quintiles over time, analyses of the IMD between 2015 and 2019 has shown this to be relatively uncommon at the extremes of deprivation (the most and least deprived deciles, when 88% and 84% respectively did not change in the time period)^[[2]](#footnote-2)^.*

**Box A1. Comparability of the Scottish and English Indices of Multiple Deprivation (SIMD and (E)IMD) .**

There are differences between the SIMD and IMD. These relate both to the spatial scale at which deprivation is measured (the population size of the spatial units used in the SIMD (c.750) is approximately half that of the those used in the IMD (c.1,500)^^[[3]](#footnote-3)^,^[[4]](#footnote-4)^^ ) and to some of the individual variables used in their construction.

Nonetheless, both indices share important similarities in terms of their fundamental composition. First, the ‘data domains’ of each are more or less identical. These are: income; employment; health; education, skills and training; crime; access to services; housing. In the SIMD, housing is a separate category, while in the IMD it is included within the ‘living environment’ domain. As an example of the similarity of the variables included within a data domain, Table A3 below compares the variables within the income domains of both indices. There are only very minor differences. Second, similar methodologies are used in the calculation of the overall indices of (area-based) relative socioeconomic deprivation, based on the ranking of the areas across all the data domains.

On account of the differences between the indices, researchers have correctly cautioned against *combining* the data within single area-based analyses spanning Great Britain^[[5]](#footnote-5)^. However, the similarities mean the two indices can be analysed separately, and results can meaningfully be assessed in terms of patterns and trends: while we cannot compare the absolute values of the two indices, the similarities of composition and methodology enable a broadly comparable understanding of the scale of, and changes to, inequalities within both countries.

**Table A3. Definitions of ‘income deprivation’ in the Scottish Index of Multiple Deprivation (2020) and (English) Index of Multiple Deprivation (IMD) 2019**

| SIMD 2020 (data all c.2017)^[[6]](#footnote-6)^ | (E)IMD 2019 (data all c.2015)^[[7]](#footnote-7)^ |
| --- | --- |
| - Number of adults receiving Income Support, income-based Employment and Support Allowance, or Jobseeker’s Allowance - Number of children (aged 0-18) dependent on a recipient of Income Support, Jobseeker’s Allowance or Employment and Support Allowance | - Adults and children in Income Support families - Adults and children in income-based Jobseeker’s Allowance families - Adults and children in income-based - Employment and Support Allowance families |
| - Number of adults receiving Guaranteed Pension Credit | - Adults and children in Pension Credit (Guarantee) families |
| - Number of people claiming Universal Credit and their dependent children (aged 0-18) (excluding those in the ‘working with no requirements’ - conditionality group) | - Adults and children in Universal Credit families where no adult is classed within the 'Working - no requirements' conditionality regime" |
| - Number of adults and children in Tax Credit families on low incomes | - Adults and children in Working Tax Credit and Child Tax Credit families not already counted, and whose equivalised income (excluding housing benefit) is below 60 per cent of the median before housing costs |
|  | - Asylum seekers in England in receipt of subsistence support, accommodation support, or both |

Figure A1. Levels of ‘income deprivation’ (defined slightly differently in England and Scotland) by city, c.2015 (England) and c.2017 (Scotland)


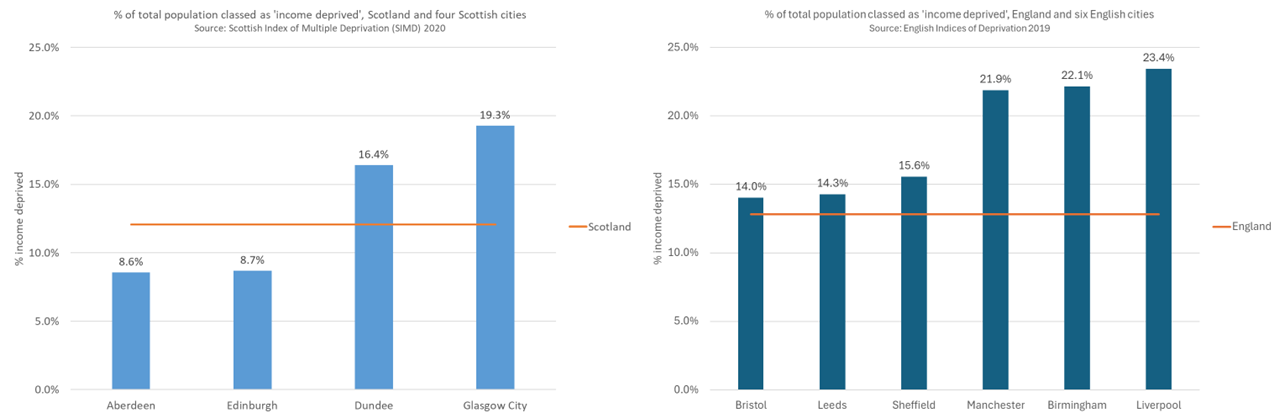


Figure A2. Levels of ‘income deprivation’ (defined slightly differently in Scotland and England) by *city-specific* quintiles, 2015 (England) and c.2017 (Scotland)
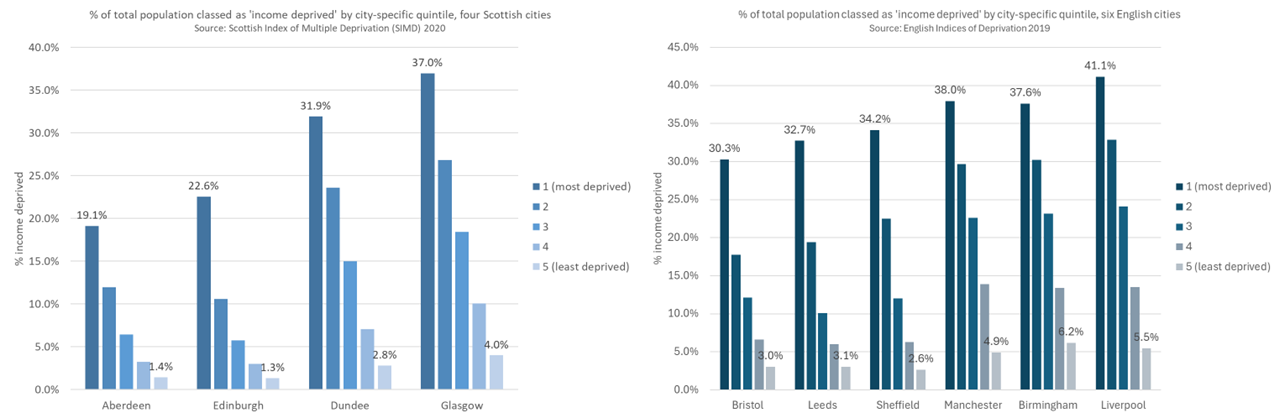


Figure A3. Age-standardised mortality rates per 100,000 population (three-year rolling averages), 1981-2020, all ages and 0-64 years: Scotland, England and their 20% most and least deprived populations – MALES


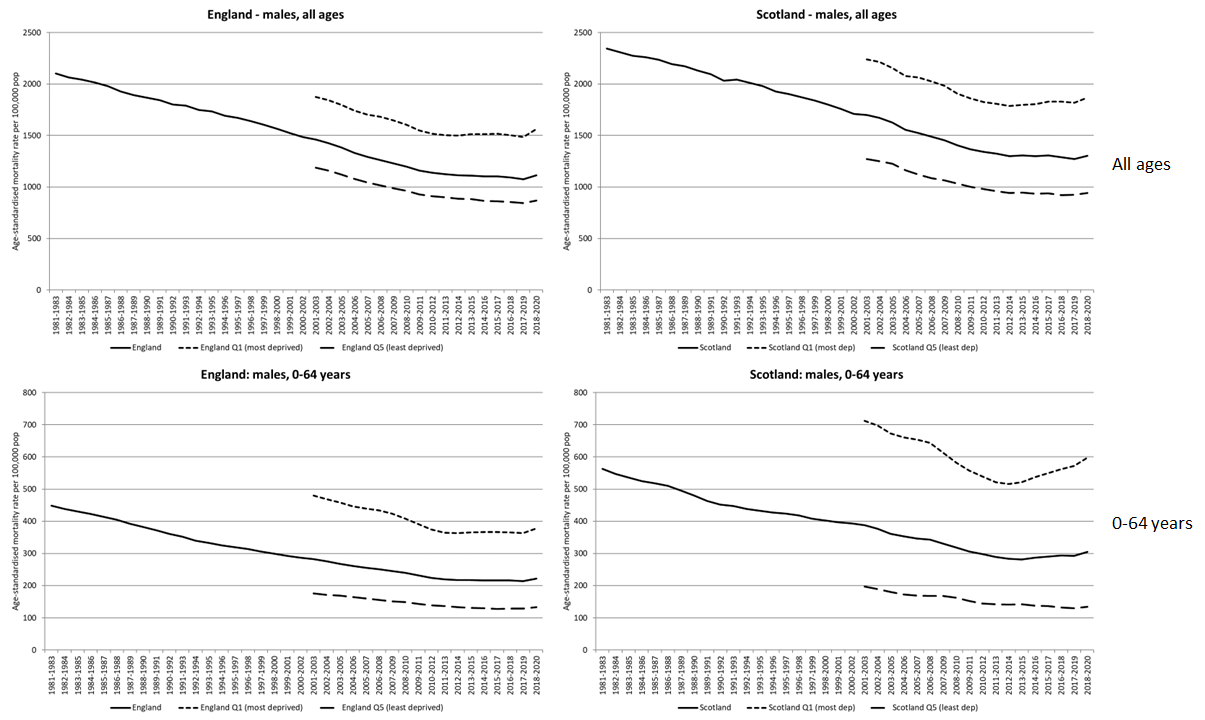


Figure A4. Age-standardised mortality rates per 100,000 population (three-year rolling averages), 1981-2020, all ages and 0-64 years: Scotland, England and their 20% most and least deprived populations – FEMALES


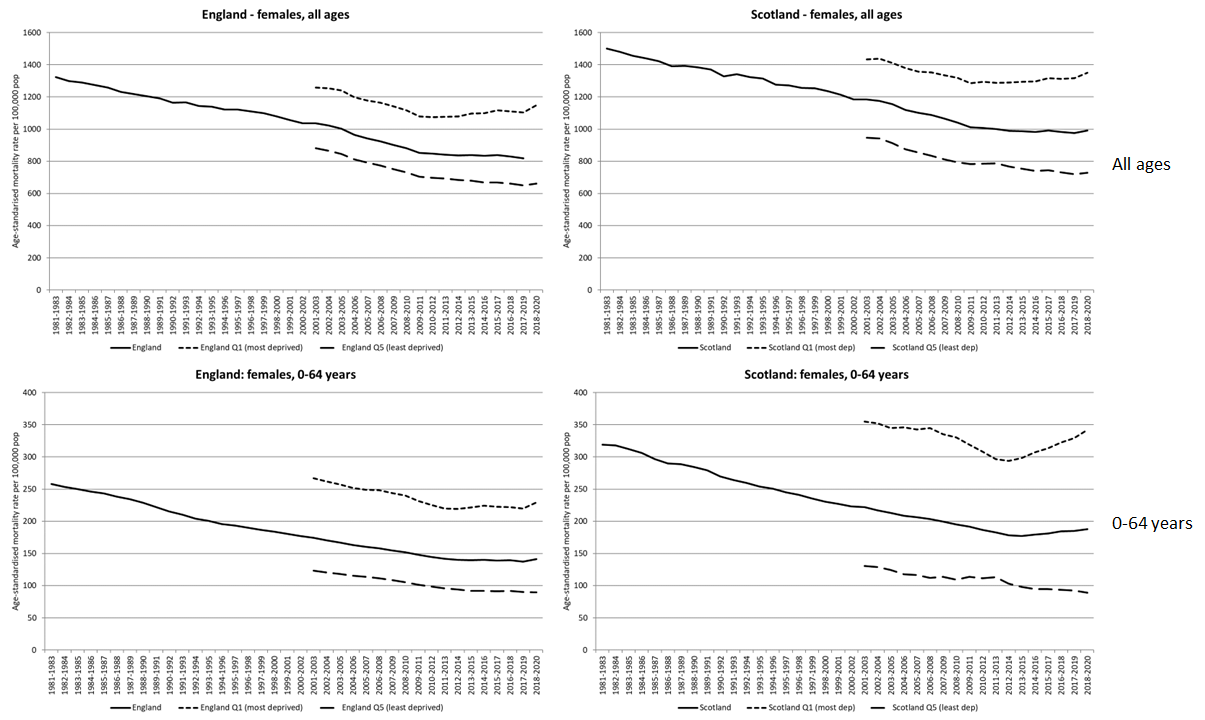


Figure A5. Age-standardised mortality rates per 100,000 population (three-year rolling averages), 1981-2020, all ages for 10 British cities and their 20% most and least deprived populations – MALES


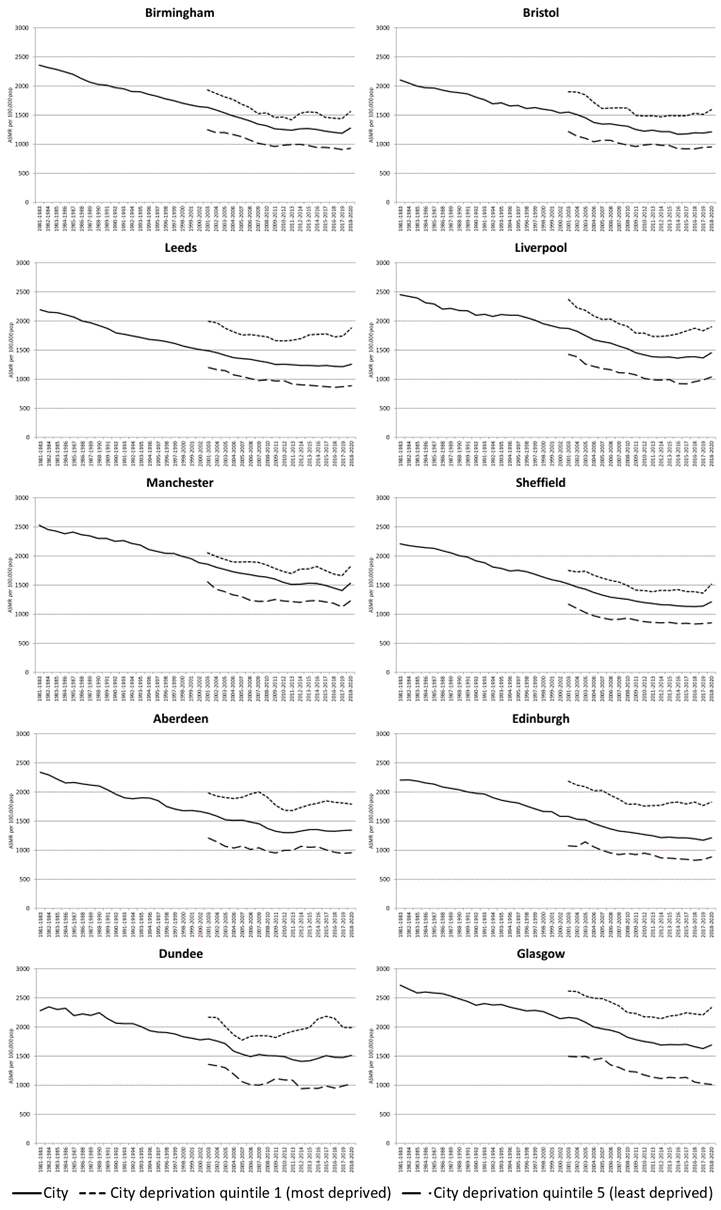
Figure A6. Age-standardised mortality rates per 100,000 population (three-year rolling averages), 1981-2020, all ages for 10 British cities and their 20% most and least deprived populations – FEMALES


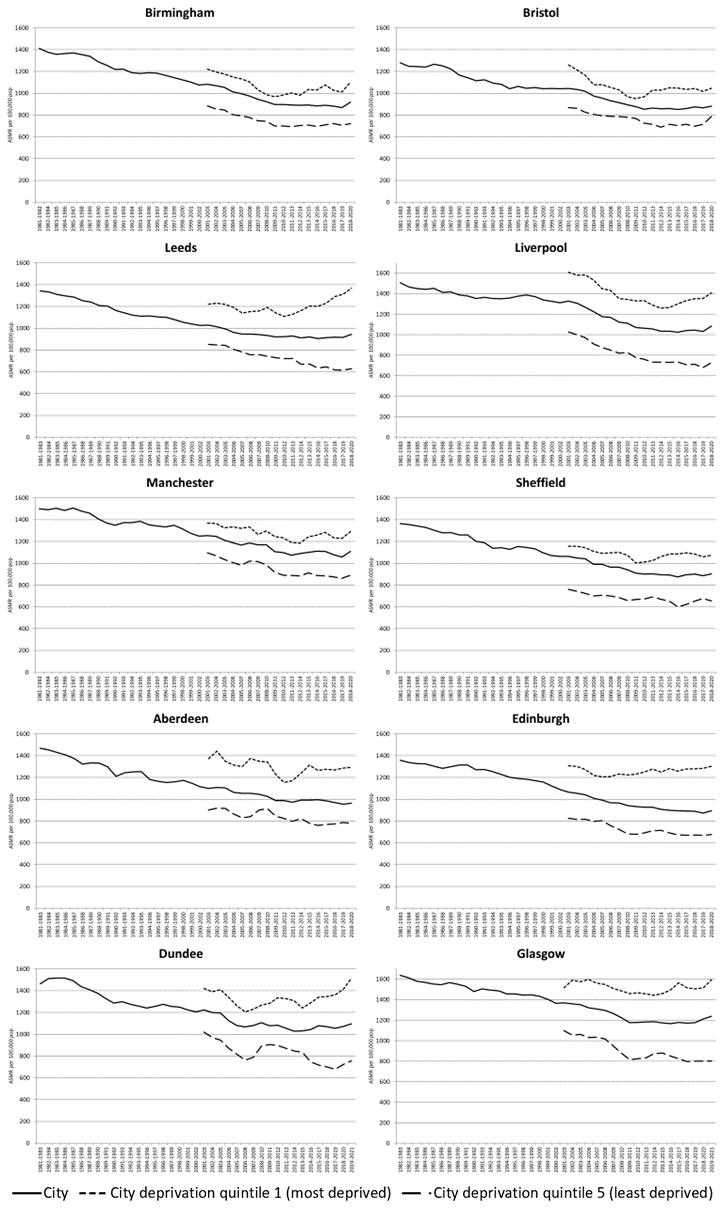


Figure A7. Age-standardised mortality rates per 100,000 population (three-year rolling averages), 1981-2020, 0-64 years, for 10 British cities and their 20% most and least deprived populations – MALES


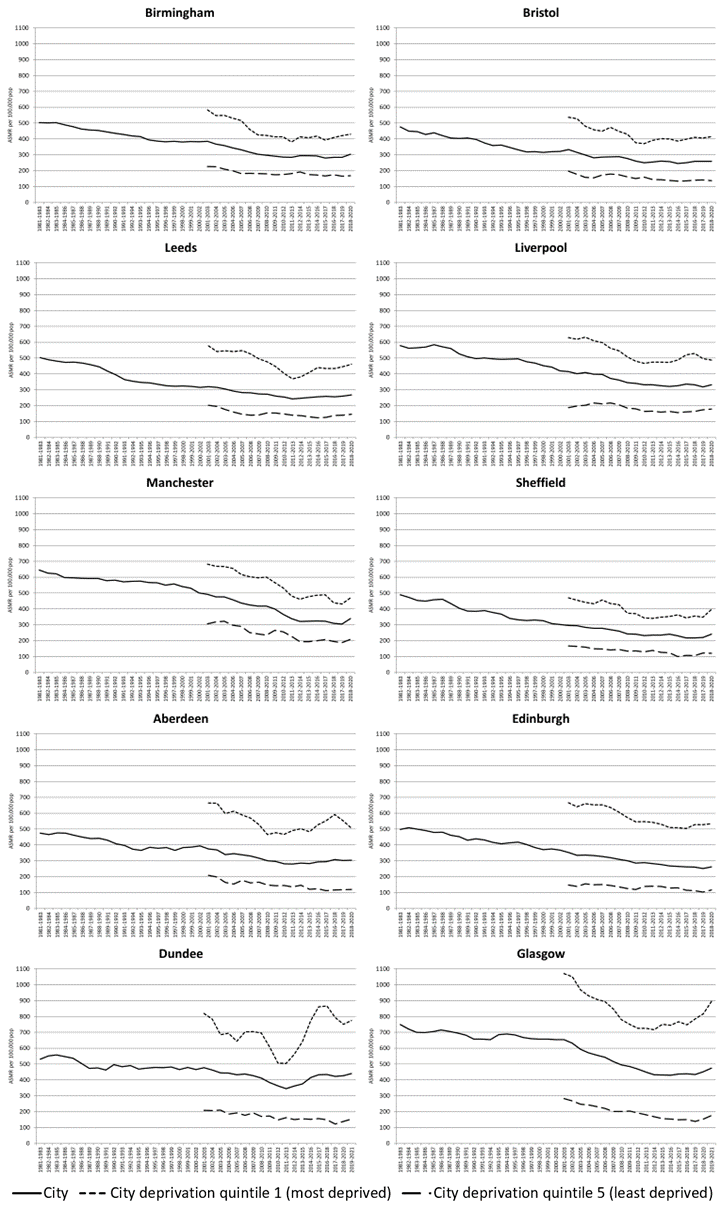


Figure A8. Age-standardised mortality rates per 100,000 population (three-year rolling averages), 1981-2020, 0-64 years, for 10 British cities and their 20% most and least deprived populations – FEMALES


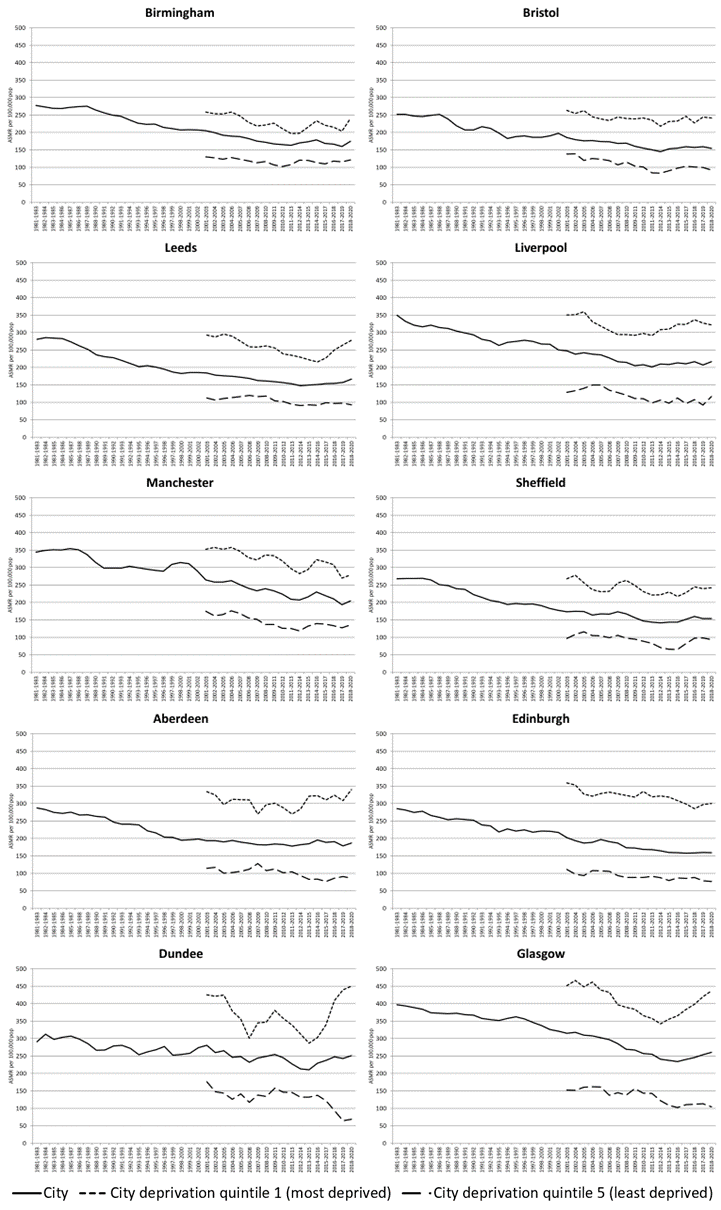


Figure A9. Trends in the Slope Index of Inequality (SII) and Relative Index of Inequality (RII) (three-year rolling averages) for age-standardised mortality rates by city-specific deprivation quintile, 1981-2020, all ages, 10 British cities


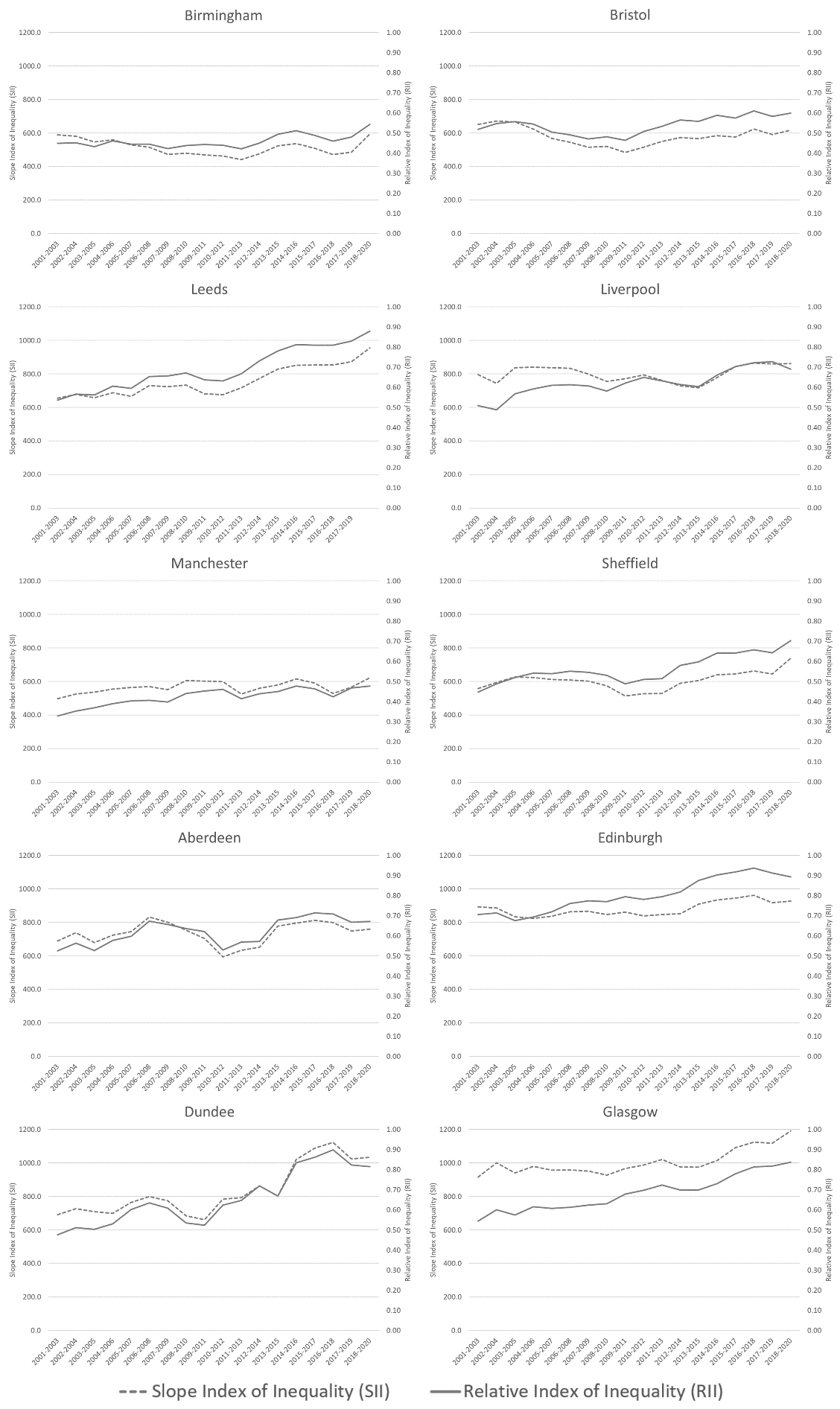


Table A4. Trends in the Slope Index of Inequality (SII) and Relative Index of Inequality (RII) for age-standardised mortality rates by city-specific deprivation quintile, 1981-2020, all ages, 10 British cities.

|  | **Glasgow** | |  | **Edinburgh** | |  | **Dundee** | |  | **Aberdeen** | |  | **Birmingham** | |  | **Bristol** |  |  | **Leeds** |  |  | **Liverpool** |  |  | **Manchester** | |  | **Sheffield** |  |
| --- | --- | --- | --- | --- | --- | --- | --- | --- | --- | --- | --- | --- | --- | --- | --- | --- | --- | --- | --- | --- | --- | --- | --- | --- | --- | --- | --- | --- | --- |
|  | **SII** | **RII** |  | **SII** | **RII** |  | **SII** | **RII** |  | **SII** | **RII** |  | **SII** | **RII** |  | **SII** | **RII** |  | **SII** | **RII** |  | **SII** | **RII** |  | **SII** | **RII** |  | **SII** | **RII** |
| 2001 | 785.4 | 0.47 |  | 879.6 | 0.68 |  | 628.6 | 0.43 |  | 604.1 | 0.46 |  | 594.0 | 0.45 |  | 616.1 | 0.50 |  | 634.0 | 0.52 |  | 1027.1 | 0.65 |  | 451.3 | 0.30 |  | 538.8 | 0.43 |
| 2002 | 1101.9 | 0.66 |  | 927.5 | 0.75 |  | 708.1 | 0.50 |  | 760.5 | 0.58 |  | 609.6 | 0.47 |  | 666.1 | 0.54 |  | 683.5 | 0.56 |  | 516.3 | 0.33 |  | 516.7 | 0.34 |  | 532.2 | 0.43 |
| 2003 | 860.5 | 0.50 |  | 872.1 | 0.69 |  | 734.5 | 0.50 |  | 705.5 | 0.54 |  | 566.2 | 0.43 |  | 673.3 | 0.52 |  | 644.9 | 0.53 |  | 843.9 | 0.54 |  | 522.2 | 0.34 |  | 605.2 | 0.49 |
| 2004 | 1039.8 | 0.64 |  | 856.6 | 0.70 |  | 740.1 | 0.53 |  | 746.9 | 0.58 |  | 569.5 | 0.46 |  | 674.9 | 0.58 |  | 703.5 | 0.61 |  | 872.3 | 0.59 |  | 536.1 | 0.37 |  | 644.4 | 0.55 |
| 2005 | 922.7 | 0.58 |  | 771.5 | 0.63 |  | 649.8 | 0.48 |  | 586.1 | 0.46 |  | 503.7 | 0.41 |  | 649.8 | 0.57 |  | 624.6 | 0.55 |  | 792.5 | 0.57 |  | 549.4 | 0.39 |  | 633.4 | 0.52 |
| 2006 | 978.8 | 0.62 |  | 845.6 | 0.74 |  | 709.2 | 0.58 |  | 839.8 | 0.69 |  | 605.8 | 0.51 |  | 546.4 | 0.49 |  | 735.1 | 0.66 |  | 855.2 | 0.62 |  | 581.0 | 0.41 |  | 595.3 | 0.55 |
| 2007 | 970.4 | 0.62 |  | 892.9 | 0.78 |  | 934.1 | 0.75 |  | 807.4 | 0.64 |  | 477.4 | 0.41 |  | 507.5 | 0.46 |  | 641.0 | 0.57 |  | 860.9 | 0.64 |  | 561.1 | 0.41 |  | 608.8 | 0.54 |
| 2008 | 924.6 | 0.60 |  | 850.1 | 0.75 |  | 752.8 | 0.58 |  | 850.8 | 0.69 |  | 464.5 | 0.41 |  | 580.4 | 0.52 |  | 817.0 | 0.72 |  | 786.0 | 0.58 |  | 566.0 | 0.40 |  | 624.5 | 0.56 |
| 2009 | 962.2 | 0.66 |  | 856.2 | 0.78 |  | 638.1 | 0.50 |  | 745.6 | 0.64 |  | 475.6 | 0.45 |  | 456.6 | 0.42 |  | 715.3 | 0.67 |  | 750.4 | 0.60 |  | 527.0 | 0.38 |  | 572.9 | 0.54 |
| 2010 | 894.3 | 0.64 |  | 833.2 | 0.77 |  | 664.4 | 0.52 |  | 661.0 | 0.58 |  | 495.2 | 0.46 |  | 521.6 | 0.50 |  | 670.1 | 0.62 |  | 726.7 | 0.57 |  | 725.8 | 0.54 |  | 527.5 | 0.50 |
| 2011 | 1041.7 | 0.74 |  | 893.0 | 0.83 |  | 679.3 | 0.55 |  | 707.2 | 0.65 |  | 438.0 | 0.43 |  | 474.6 | 0.47 |  | 659.2 | 0.62 |  | 835.9 | 0.69 |  | 554.3 | 0.44 |  | 439.9 | 0.43 |
| 2012 | 1026.6 | 0.72 |  | 791.8 | 0.74 |  | 1011.1 | 0.80 |  | 411.6 | 0.36 |  | 454.4 | 0.43 |  | 551.3 | 0.56 |  | 699.2 | 0.65 |  | 818.3 | 0.69 |  | 516.6 | 0.41 |  | 612.5 | 0.60 |
| 2013 | 992.1 | 0.71 |  | 856.3 | 0.81 |  | 685.4 | 0.60 |  | 779.1 | 0.70 |  | 430.4 | 0.40 |  | 621.1 | 0.57 |  | 790.7 | 0.73 |  | 630.8 | 0.51 |  | 502.2 | 0.40 |  | 533.0 | 0.51 |
| 2014 | 910.2 | 0.67 |  | 907.7 | 0.90 |  | 890.6 | 0.76 |  | 763.1 | 0.66 |  | 542.3 | 0.51 |  | 543.7 | 0.56 |  | 829.7 | 0.81 |  | 735.5 | 0.64 |  | 661.3 | 0.51 |  | 622.3 | 0.63 |
| 2015 | 1021.9 | 0.71 |  | 965.5 | 0.92 |  | 829.1 | 0.65 |  | 792.0 | 0.68 |  | 599.7 | 0.56 |  | 535.1 | 0.54 |  | 865.5 | 0.80 |  | 786.4 | 0.66 |  | 573.4 | 0.44 |  | 662.3 | 0.65 |
| 2016 | 1110.0 | 0.81 |  | 926.0 | 0.89 |  | 1342.2 | 1.09 |  | 834.8 | 0.74 |  | 467.9 | 0.46 |  | 674.2 | 0.66 |  | 857.9 | 0.82 |  | 814.0 | 0.68 |  | 612.7 | 0.48 |  | 634.9 | 0.64 |
| 2017 | 1142.4 | 0.81 |  | 943.9 | 0.94 |  | 1090.9 | 0.84 |  | 811.9 | 0.73 |  | 460.3 | 0.44 |  | 518.5 | 0.52 |  | 838.7 | 0.81 |  | 928.5 | 0.77 |  | 588.3 | 0.47 |  | 635.5 | 0.63 |
| 2018 | 1119.8 | 0.82 |  | 1012.7 | 0.98 |  | 934.8 | 0.76 |  | 750.0 | 0.66 |  | 487.2 | 0.48 |  | 676.4 | 0.65 |  | 860.9 | 0.80 |  | 853.5 | 0.71 |  | 384.4 | 0.32 |  | 718.5 | 0.70 |
| 2019 | 1092.3 | 0.82 |  | 797.1 | 0.82 |  | 1044.1 | 0.87 |  | 681.5 | 0.61 |  | 507.7 | 0.52 |  | 579.3 | 0.58 |  | 918.4 | 0.88 |  | 798.9 | 0.70 |  | 727.1 | 0.61 |  | 580.7 | 0.59 |
| 2020 | 1359.7 | 0.87 |  | 971.8 | 0.88 |  | 1123.0 | 0.82 |  | 847.1 | 0.74 |  | 789.1 | 0.64 |  | 597.8 | 0.57 |  | 1086.5 | 0.95 |  | 931.0 | 0.66 |  | 752.6 | 0.50 |  | 916.8 | 0.81 |

Figure A10. Age-standardised mortality rates per 100,000 population (three-year rolling averages), 1981-2020, all ages and 0-64 years: Scotland, England and their 20% most and least deprived populations - *including 95% confidence intervals.*


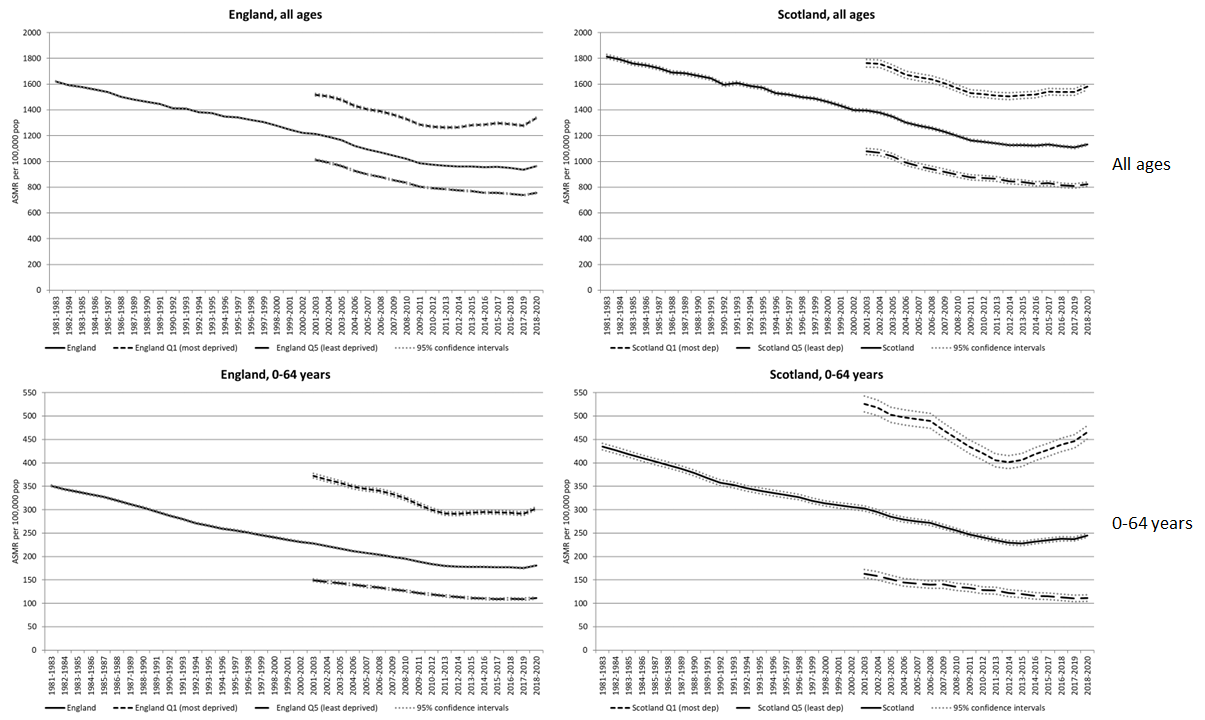


Figure A11. Age-standardised mortality rates per 100,000 population (three-year rolling averages), 1981-2020, all ages, for 10 British cities and their 20% most and least deprived populations - *including 95% confidence intervals.*


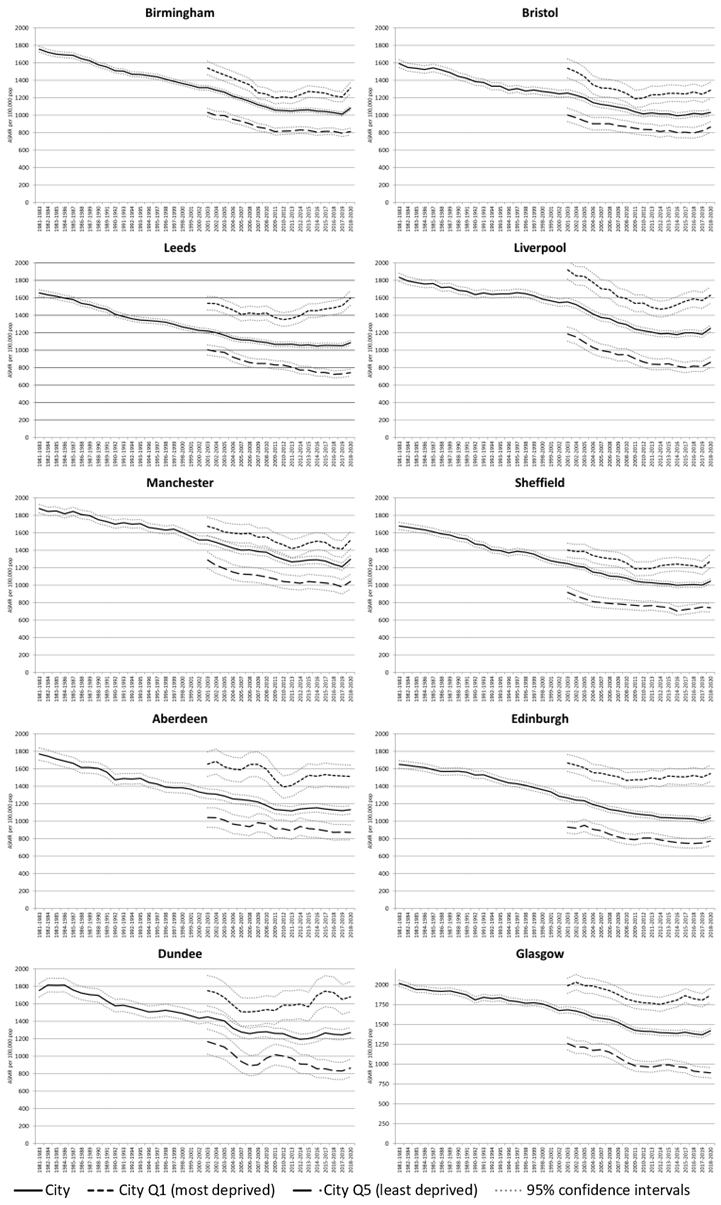


Figure A12. Age-standardised mortality rates per 100,000 population (three-year rolling averages), 1981-2020, 0-64 years, for 10 British cities and their 20% most and least deprived populations - *including 95% confidence intervals.*


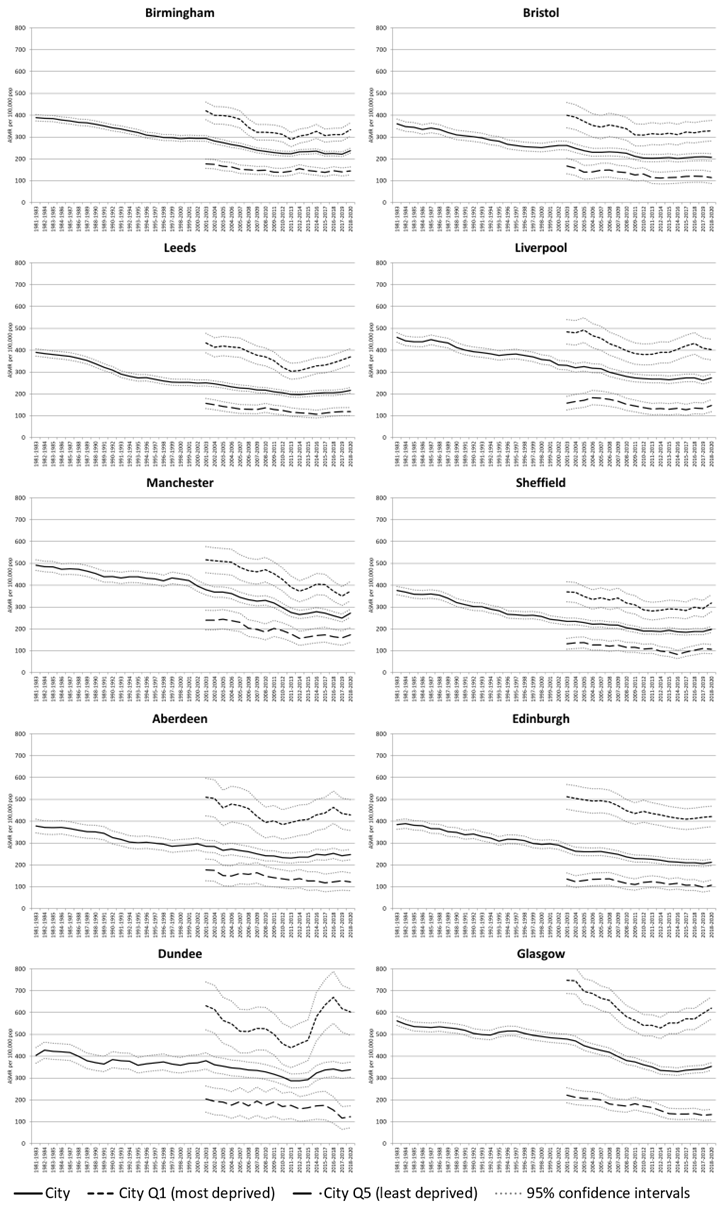


Figure A13. Trends in the Slope Index of Inequality (SII) and Relative Index of Inequality (RII) for age-standardised mortality rates by deprivation quintile, 1981-2020, all ages, Scotland and England - *including 95% confidence intervals.*


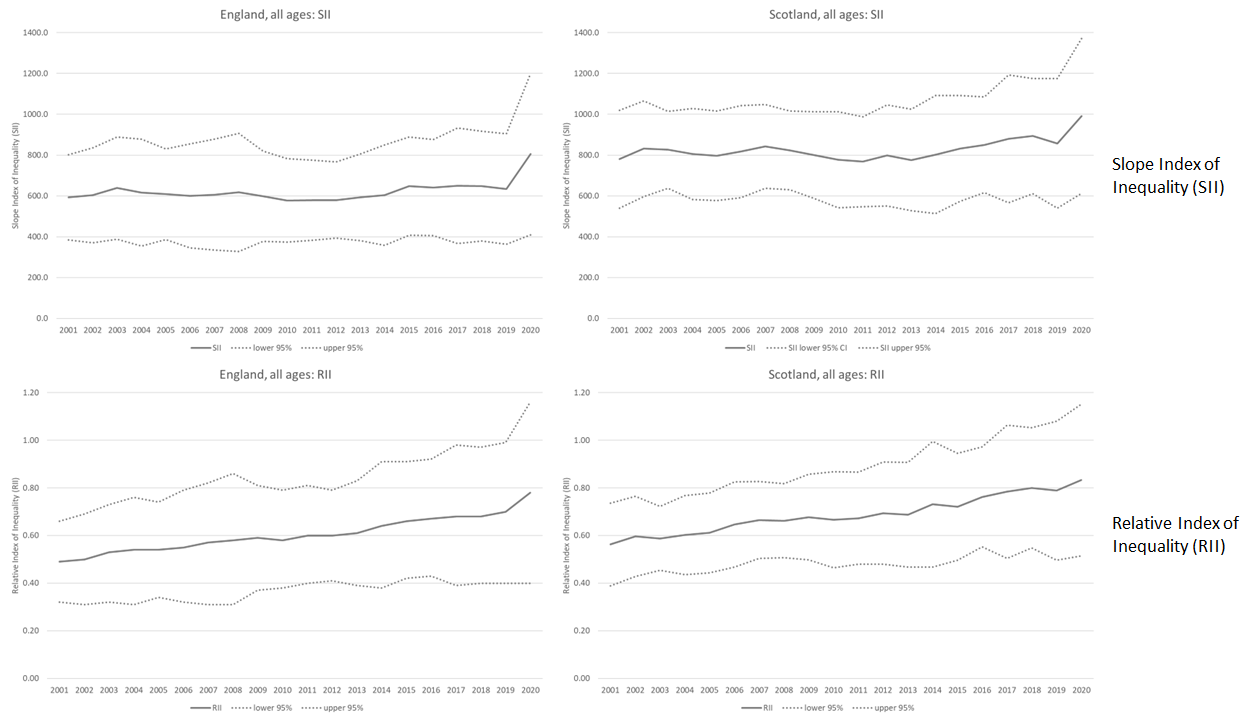


Figure A14. Trends in the Slope Index of Inequality (SII) and Relative Index of Inequality (RII) for age-standardised mortality rates by deprivation quintile, 1981-2020, 0-64 years, Scotland and England - *including 95% confidence intervals.*


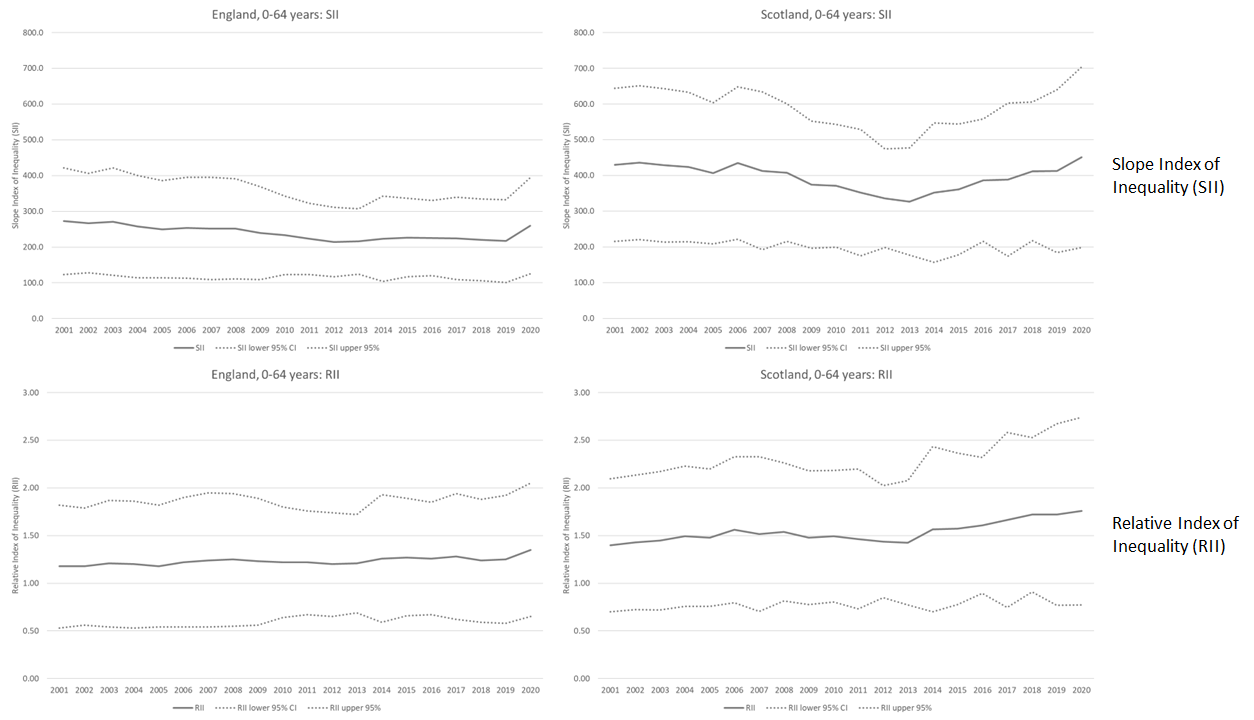


Table A5 (a). Trends in the Slope Index of Inequality (SII) and Relative Index of Inequality (RII) for age-standardised mortality rates by city-specific deprivation quintile, 1981-2020, all ages, 6 English cities - *including 95% confidence intervals*.

|  | **Birmingham** |  | **Bristol** |  | **Leeds** |  | **Liverpool** |  | **Manchester** |  | **Sheffield** |  |
| --- | --- | --- | --- | --- | --- | --- | --- | --- | --- | --- | --- | --- |
|  | **SII (95% CIs)** | **RII (95%CIs)** | **SII (95% CIs)** | **RII (95%CIs)** | **SII (95% CIs)** | **RII (95%CIs)** | **SII (95% CIs)** | **RII (95%CIs)** | **SII (95% CIs)** | **RII (95%CIs)** | **SII (95% CIs)** | **RII (95%CIs)** |
| 2001 | 594.0 (371.6-816.4) | 0.45 (0.28-0.62) | 616.1 (248.1-984.1) | 0.50 (0.20-0.79) | 634.0 (386.0-881.9) | 0.52 (0.32-0.72) | 1027.1 (391.1-1663.1) | 0.65 (0.25-1.06) | 451.3 (-66.3-968.9) | 0.30 (-0.04-0.63) | 538.8 (164.4-913.2) | 0.43 (0.13-0.73) |
| 2002 | 609.6 (204.9-1014.2) | 0.47 (0.16-0.78) | 666.1 (434.1-898.0) | 0.54 (0.35-0.72) | 683.5 (574.5-792.5) | 0.56 (0.47-0.65) | 516.3 (-420.7-1453.2) | 0.33 (-0.27-0.93) | 516.7 (221.0-812.5) | 0.34 (0.15-0.54) | 532.2 (-107-1171.5) | 0.43 (-0.09-0.94) |
| 2003 | 566.2 (208.4-924.0) | 0.43 (0.16-0.70) | 673.3 (491.0-855.6) | 0.52 (0.38-0.66) | 644.9 (350.5-939.2) | 0.53 (0.29-0.77) | 843.9 (156.5-1531.3) | 0.54 (0.10-0.98) | 522.2 (234.4-810.0) | 0.34 (0.15-0.53) | 605.2 (-88.6-1299.0) | 0.49 (-0.07-1.05) |
| 2004 | 569.5 (110.2-1028.8) | 0.46 (0.09-0.83) | 674.9 (534.6-815.2) | 0.58 (0.46-0.70) | 703.5 (307.4-1099.6) | 0.61 (0.27-0.95) | 872.3 (159.1-1585.4) | 0.59 (0.11-1.07) | 536.1 (11.8-1060.3) | 0.37 (0.01-0.74) | 644.4 (236.0-1052.8) | 0.55 (0.20-0.90) |
| 2005 | 503.7 (200.9-806.4) | 0.41 (0.16-0.66) | 649.8 (501.3-798.2) | 0.57 (0.44-0.69) | 624.6 (414.7-834.4) | 0.55 (0.36-0.73) | 792.5 (138.6-1446.4) | 0.57 (0.10-1.04) | 549.4 (216.5-882.4) | 0.39 (0.15-0.63) | 633.4 (-6.4-1273.3) | 0.52 (-0.01-1.05) |
| 2006 | 605.8 (341.5-870.2) | 0.51 (0.29-0.73) | 546.4 (-5.5-1098.2) | 0.49 (0.00-0.98) | 735.1 (679.6-790.6) | 0.66 (0.61-0.71) | 855.2 (557.9-1152.5) | 0.62 (0.40-0.83) | 581.0 (304.1-857.8) | 0.41 (0.21-0.60) | 595.3 (191.7-999.0) | 0.55 (0.18-0.93) |
| 2007 | 477.4 (-37.7-992.5) | 0.41 (-0.03-0.86) | 507.5 (418.8-596.2) | 0.46 (0.38-0.54) | 641.0 (526.7-755.2) | 0.57 (0.47-0.68) | 860.9 (398.9-1322.8) | 0.64 (0.30-0.99) | 561.1 (266.1-856.2) | 0.41 (0.19-0.63) | 608.8 (204.4-1013.2) | 0.54 (0.18-0.90) |
| 2008 | 464.5 (67.4-861.7) | 0.41 (0.06-0.76) | 580.4 (406.5-754.3) | 0.52 (0.37-0.68) | 817.0 (638.4-995.5) | 0.72 (0.56-0.88) | 786.0 (262.6-1309.4) | 0.58 (0.19-0.96) | 566.0 (82.7-1049.3) | 0.40 (0.06-0.74) | 624.5 (168.9-1080.2) | 0.56 (0.15-0.97) |
| 2009 | 475.6 (-47.6-998.7) | 0.45 (-0.04-0.94) | 456.6 (-13.4-926.6) | 0.42 (-0.01-0.86) | 715.3 (614.5-816.0) | 0.67 (0.58-0.77) | 750.4 (361.3-1139.6) | 0.60 (0.29-0.91) | 527.0 (95.3-958.7) | 0.38 (0.07-0.70) | 572.9 (97.2-1048.7) | 0.54 (0.09-0.98) |
| 2010 | 495.2 (145.5-844.9) | 0.46 (0.13-0.78) | 521.6 (25.3-1017.8) | 0.50 (0.02-0.97) | 670.1 (364.5-975.8) | 0.62 (0.34-0.90) | 726.7 (364.9-1088.6) | 0.57 (0.28-0.85) | 725.8 (324.7-1126.9) | 0.54 (0.24-0.83) | 527.5 (80.6-974.5) | 0.50 (0.08-0.92) |
| 2011 | 438.0 (8.3-867.8) | 0.43 (0.01-0.84) | 474.6 (273.8-675.4) | 0.47 (0.27-0.67) | 659.2 (413.7-904.8) | 0.62 (0.39-0.85) | 835.9 (357.3-1314.4) | 0.69 (0.30-1.09) | 554.3 (146.6-961.9) | 0.44 (0.12-0.76) | 439.9 (-49.1-929.0) | 0.43 (-0.05-0.91) |
| 2012 | 454.4 (130.1-778.6) | 0.43 (0.12-0.74) | 551.3 (361.5-741.2) | 0.56 (0.37-0.75) | 699.2 (404.7-993.7) | 0.65 (0.38-0.93) | 818.3 (222.3-1414.2) | 0.69 (0.19-1.19) | 516.6 (58.9-974.4) | 0.41 (0.05-0.77) | 612.5 (257.8-967.2) | 0.60 (0.25-0.95) |
| 2013 | 430.4 (90.1-770.7) | 0.40 (0.08-0.72) | 621.1 (242-1000.2) | 0.57 (0.22-0.93) | 790.7 (719.2-862.2) | 0.73 (0.66-0.79) | 630.8 (18.7-1242.9) | 0.51 (0.02-1.01) | 502.2 (187.2-817.1) | 0.40 (0.15-0.65) | 533.0 (117.7-948.4) | 0.51 (0.11-0.91) |
| 2014 | 542.3 (231.8-852.9) | 0.51 (0.22-0.81) | 543.7 (402.1-685.3) | 0.56 (0.41-0.71) | 829.7 (606.3-1053.1) | 0.81 (0.60-1.03) | 735.5 (399.5-1071.4) | 0.64 (0.35-0.93) | 661.3 (404.7-917.9) | 0.51 (0.31-0.71) | 622.3 (418-826.6) | 0.63 (0.42-0.84) |
| 2015 | 599.7 (309.1-890.3) | 0.56 (0.29-0.84) | 535.1 (208.7-861.5) | 0.54 (0.21-0.87) | 865.5 (718.1-1012.9) | 0.80 (0.66-0.94) | 786.4 (97.1-1475.6) | 0.66 (0.08-1.23) | 573.4 (260.0-886.8) | 0.44 (0.20-0.68) | 662.3 (356.5-968.1) | 0.65 (0.35-0.96) |
| 2016 | 467.9 (77.3-858.5) | 0.46 (0.08-0.84) | 674.2 (565.4-782.9) | 0.66 (0.56-0.77) | 857.9 (660.7-1055.1) | 0.82 (0.63-1.01) | 814.0 (251.5-1376.5) | 0.68 (0.21-1.16) | 612.7 (258.2-967.2) | 0.48 (0.20-0.76) | 634.9 (279.9-989.9) | 0.64 (0.28-1.00) |
| 2017 | 460.3 (306.8-613.7) | 0.44 (0.30-0.59) | 518.5 (280.1-757.0) | 0.52 (0.28-0.76) | 838.7 (383.4-1294.0) | 0.81 (0.37-1.24) | 928.5 (634.6-1222.4) | 0.77 (0.53-1.01) | 588.3 (180.2-996.4) | 0.47 (0.14-0.80) | 635.5 (303.2-967.8) | 0.63 (0.30-0.96) |
| 2018 | 487.2 (278.0-696.4) | 0.48 (0.27-0.68) | 676.4 (490.8-862.0) | 0.65 (0.47-0.82) | 860.9 (348.4-1373.3) | 0.08 (0.33-1.28) | 853.5 (410.6-1296.4) | 0.71 (0.34-1.08) | 384.4 (76.5-692.3) | 0.32 (0.06-0.58) | 718.5 (275.2-1161.8) | 0.7 (0.27-1.14) |
| 2019 | 507.7 (137.4-878.1) | 0.52 (0.14-0.89) | 579.3 (163.5-995.0) | 0.58 (0.17-1.00) | 918.4 (548.7-1288.0) | 0.88 (0.53-1.24) | 798.9 (498.9-1099.0) | 0.70 (0.44-0.96) | 727.1 (640.3-814.0) | 0.61 (0.54-0.68) | 580.7 (196-965.3) | 0.59 (0.20-0.99) |
| 2020 | 789.1 (488.2-1090.0) | 0.64 (0.39-0.88) | 597.8 (22.1-1173.6) | 0.57 (0.02-1.11) | 1086.5 (421.1-1751.8) | 0.95 (0.37-1.53) | 931.0 (335.0-1527.0) | 0.66 (0.24-1.08) | 752.6 (632.3-872.9) | 0.50 (0.42-0.58) | 916.8 (502.9-1330.8) | 0.81 (0.44-1.18) |

Table A5 (b). Trends in the Slope Index of Inequality (SII) and Relative Index of Inequality (RII) for age-standardised mortality rates by city-specific deprivation quintile, 1981-2020, all ages, 4 Scottish cities - *including 95% confidence intervals*.

|  | Glasgow |  |  | Edinburgh |  |  | Dundee |  |  | Aberdeen |  |
| --- | --- | --- | --- | --- | --- | --- | --- | --- | --- | --- | --- |
|  | SII (95% CIs) | RII (95%CIs) |  | SII (95% CIs) | RII (95%CIs) |  | SII (95% CIs) | RII (95%CIs) |  | SII (95% CIs) | RII (95%CIs) |
| 2001 | 785.4 (215.9-1355.0) | 0.47 (0.13-0.81) |  | 879.6 (570.9-1188.3) | 0.68 (0.44-0.92) |  | 628.6 (68.3-1189.0) | 0.43 (0.05-0.81) |  | 604.1 (289.2-919.1) | 0.46 (0.22-0.70) |
| 2002 | 1101.9 (834.5-1369.2) | 0.66 (0.50-0.82) |  | 927.5 (548.5-1306.6) | 0.75 (0.44-1.05) |  | 708.1 (442.1-974.1) | 0.50 (0.31-0.68) |  | 760.5 (374.6-1146.4) | 0.58 (0.28-0.87) |
| 2003 | 860.5 (480.5-1240.4) | 0.50 (0.28-0.72) |  | 872.1 (279.3-1465.0) | 0.69 (0.22-1.16) |  | 734.5 (593.7-875.4) | 0.50 (0.40-0.60) |  | 705.5 (82.8-1328.2) | 0.54 (0.06-1.01) |
| 2004 | 1039.8 (680.2-1399.4) | 0.64 (0.42-0.86) |  | 856.6 (599.0-1114.2) | 0.7 (0.49-0.92) |  | 740.1 (402.9-1077.3) | 0.53 (0.29-0.78) |  | 746.9 (357.6-1136.2) | 0.58 (0.28-0.88) |
| 2005 | 922.7 (711.3-1134.1) | 0.58 (0.45-0.71) |  | 771.5 (525.6-1017.5) | 0.63 (0.43-0.83) |  | 649.8 (286.6-1013.0) | 0.48 (0.21-0.74) |  | 586.1 (270.3-901.9) | 0.46 (0.21-0.71) |
| 2006 | 978.8 (861.3-1096.4) | 0.62 (0.55-0.70) |  | 845.6 (567.7-1123.6) | 0.74 (0.50-0.99) |  | 709.2 (562.2-856.1) | 0.58 (0.46-0.7) |  | 839.8 (481.0-1198.7) | 0.69 (0.39-0.98) |
| 2007 | 970.4 (757.5-1183.2) | 0.62 (0.48-0.75) |  | 892.9 (430.1-1355.8) | 0.78 (0.38-1.19) |  | 934.1 (-4.7-1872.9) | 0.75 (0.00-1.50) |  | 807.4 (541.3-1073.6) | 0.64 (0.43-0.85) |
| 2008 | 924.6 (575.6-1273.5) | 0.60 (0.37-0.82) |  | 850.1 (599.2-1101.1) | 0.75 (0.53-0.98) |  | 752.8 (238.3-1267.4) | 0.58 (0.18-0.97) |  | 850.8 (113.1-1588.6) | 0.69 (0.09-1.29) |
| 2009 | 962.2 (547.3-1377.0) | 0.66 (0.37-0.94) |  | 856.2 (687.8-1024.6) | 0.78 (0.63-0.94) |  | 638.1 (384.0-892.2) | 0.50 (0.30-0.70) |  | 745.6 (15.9-1475.3) | 0.64 (0.01-1.26) |
| 2010 | 894.3 (607.5-1181.1) | 0.64 (0.43-0.84) |  | 833.2 (679.4-987.0) | 0.77 (0.63-0.92) |  | 664.4 (213.3-1115.4) | 0.52 (0.17-0.88) |  | 661.0 (382.4-939.5) | 0.58 (0.34-0.83) |
| 2011 | 1041.7 (903.7-1179.6) | 0.74 (0.64-0.84) |  | 893.0 (503.6-1282.4) | 0.83 (0.47-1.19) |  | 679.3 (362.3-996.3) | 0.55 (0.29-0.80) |  | 707.2 (427.3-987.2) | 0.65 (0.39-0.90) |
| 2012 | 1026.6 (764.4-1288.8) | 0.72 (0.53-0.90) |  | 791.8 (469.5-1114.1) | 0.74 (0.44-1.05) |  | 1011.1 (767.8-1254.4) | 0.80 (0.61-0.99) |  | 411.6 (170.8-652.5) | 0.36 (0.15-0.57) |
| 2013 | 992.1 (819.1-1165) | 0.71 (0.59-0.84) |  | 856.3 (308.4-1404.2) | 0.81 (0.29-1.33) |  | 685.4 (466.5-904.4) | 0.60 (0.41-0.79) |  | 779.1 (321.7-1236.6) | 0.70 (0.29-1.11) |
| 2014 | 910.2 (776.6-1043.7) | 0.67 (0.57-0.77) |  | 907.7 (478.1-1337.4) | 0.90 (0.47-1.32) |  | 890.6 (693.0-1088.2) | 0.76 (0.59-0.93) |  | 763.1 (469.1-1057.2) | 0.66 (0.40-0.91) |
| 2015 | 1021.9 (730.1-1313.7) | 0.71 (0.51-0.92) |  | 965.5 (499.1-1431.8) | 0.92 (0.47-1.36) |  | 829.1 (489.9-1168.3) | 0.65 (0.38-0.91) |  | 792 (495.6-1088.3) | 0.68 (0.42-0.93) |
| 2016 | 1110.0 (940.4-1279.5) | 0.81 (0.69-0.93) |  | 926.0 (643.2-1208.8) | 0.89 (0.62-1.17) |  | 1342.2 (998.5-1685.9) | 1.09 (0.81-1.37) |  | 834.8 (625.4-1044.3) | 0.74 (0.55-0.92) |
| 2017 | 1142.4 (875.4-1409.3) | 0.81 (0.62-1.01) |  | 943.9 (532.9-1354.8) | 0.94 (0.53-1.35) |  | 1090.9 (699.9-1481.8) | 0.84 (0.54-1.15) |  | 811.9 (333.4-1290.4) | 0.73 (0.30-1.15) |
| 2018 | 1119.8 (652.7-1586.9) | 0.82 (0.48-1.16) |  | 1012.7 (385.4-1640) | 0.98 (0.37-1.58) |  | 934.8 (662.4-1207.1) | 0.76 (0.54-0.98) |  | 750.0 (500.8-999.2) | 0.66 (0.44-0.88) |
| 2019 | 1092.3 (805.9-1378.6) | 0.82 (0.60-1.03) |  | 797.1 (259.4-1334.9) | 0.82 (0.27-1.37) |  | 1044.1 (626.7-1461.5) | 0.87 (0.52-1.21) |  | 681.5 (259.4-1103.7) | 0.61 (0.23-0.99) |
| 2020 | 1359.7 (1120.1-1599.3) | 0.87 (0.72-1.03) |  | 971.8 (563.3-1380.4) | 0.88 (0.51-1.26) |  | 1123.0 (812.4-1433.6) | 0.82 (0.59-1.05) |  | 847.1 (200.7-1493.4) | 0.74 (0.17-1.30) |

**Additional references for article**

*As the journal has a maximum number of 40 references, some had to be excluded from the main manuscript, but are included here.*

**Introduction**:

- Poverty rates are higher in urban areas than in rural areas across the nations of the UK.^[[8]](#endnote-1)^,^[[9]](#endnote-2)^^[[10]](#endnote-3)-^^[[11]](#endnote-4)^

**Introduction**:

- Cuts to social security and local government services have been greatest in more deprived areas.^[[12]](#endnote-5),^^[[13]](#endnote-6)^

*These references:*

1. Source: Office for National Statistics (ONS). Dataset: estimates of the population for the UK, England, Wales, Scotland, and Northern Ireland. Available from: <https://www.ons.gov.uk/peoplepopulationandcommunity/populationandmigration/populationestimates/datasets/populationestimatesforukenglandandwalesscotlandandnorthernireland> (Accessed October 2024) [↑](#footnote-ref-1)
2. Source: McLennan D., Noble S., Noble M. et al. The English Indices of Deprivation 2019 – technical report. London: UK Government Ministry of Housing, Communities & Local Government; 2019 [↑](#footnote-ref-2)
3. Schofield L., Walsh D., Munoz-Arroyo R., McCartney G., Buchanan D., Lawder R., Armstrong M., Dundas R., H Leyland A.H. Dying younger in Scotland: trends in mortality and deprivation relative to England and Wales, 1981-2011. Health & Place 2016; 40: 106-115 [↑](#footnote-ref-3)
4. Walsh D., Bendel N., Jones R., Hanlon P. It’s not ‘just deprivation’: Why do equally deprived UK cities experience different health outcomes? Public Health 2010; 124: 487-495 [↑](#footnote-ref-4)
5. Abel GA, Barclay ME, Payne RA. Adjusted indices of multiple deprivation to enable comparisons within and between constituent countries of the UK including an illustration using mortality rates. BMJ Open 2016; 6(11): e012750 [↑](#footnote-ref-5)
6. Source: Scottish Government (SG). SIMD 2020 technical notes. Edinburgh: SG; 2020 [↑](#footnote-ref-6)
7. Source: McLennan D., Noble S., Noble M. et al. The English Indices of Deprivation 2019 – technical report. London: UK Government Ministry of Housing, Communities & Local Government; 2019 [↑](#footnote-ref-7)
8. McCartney G, Hoggett R. How well does the Scottish Index of Multiple Deprivation identify income and employment deprived individuals across the urban-rural spectrum and between local authorities? Public Health 2023; 217: 26-32. [↑](#endnote-ref-1)
9. Vera-Toscano, E., Shucksmith, M., Brown, D. L., Brown, H. The rural–urban poverty gap in England after the 2008 financial crisis: exploring the effects of budgetary cuts and welfare reforms. Regional Studies 2023; 58(6): 1264–1281. [↑](#endnote-ref-2)
10. Devlin A., McKay K., Russell R.. Multiple Deprivation in Northern Ireland. Belfast: Northern Ireland Assembly; 2018 [↑](#endnote-ref-3)
11. Jones L. Welsh Index of Multiple Deprivation 2014: A guide to analysing deprivation in rural areas. Cardiff: Welsh Government; 2015 [↑](#endnote-ref-4)
12. Gray M, Barford A. The depths of the cuts: the uneven geography of local government austerity. Cambridge Journal of Regions, Economy and Society 2018; 11: 541–63. [↑](#endnote-ref-5)
13. Beatty C, Fothergill S. The uneven impact of welfare reform: the financial losses to places and people. Sheffield: Sheffield Hallam, 2016 [↑](#endnote-ref-6)
